# Hyperbaric Oxygen Therapy to Minimize Orthodontic Relapse in Rabbits

**DOI:** 10.1055/s-0043-1776118

**Published:** 2024-01-10

**Authors:** Yun Mukmin Akbar, Ani Melani Maskoen, Endah Mardiati, Ganesha Wandawa, Amaliya Amaliya, Ida Bagus Narmada, Nina Djustiana, Ida Ayu Evangelina, Rasmi Rikmasari, Mas Rizky Anggun

**Affiliations:** 1 Faculty of Dentistry, Universitas Padjadjaran, Bandung, Indonesia; 2 Department of Research and Development, Indonesian Naval Dental Institute R.E. Martadinata, Jakarta, Indonesia; 3 Department of Oral Biology, Faculty of Dentistry, Universitas Padjadjaran, Bandung, Indonesia; 4 Department of Orthodontics, Faculty of Dentistry, Universitas Padjadjaran, Bandung, Indonesia; 5 Departement of Periodontology, Faculty of Dentistry, Universitas Padjadjaran, Bandung, Indonesia; 6 Center Study for Military Dentistry, Faculty of Dentistry, Universitas Padjadjaran, Bandung, Indonesia; 7 Department of Orthodontics, Faculty of Dentistry, Universitas Airlangga, Surabaya, Indonesia; 8 Department of Dental Material, Faculty of Dentistry, Universitas Padjadjaran, Bandung, Indonesia; 9 Department of Prosthodontics, Faculty of Dentistry, Universitas Padjadjaran, Bandung, Indonesia; 10 Department of Biomedical Sciences, Faculty of Medicine, Universitas Padjadjaran, Bandung, Indonesia

**Keywords:** hyperbaric oxygen therapy, orthodontic relapse, collagen synthesis and degradation, supracrestal gingival fibers, animal model

## Abstract

**Objectives** The purpose of the present study was to discover how hyperbaric oxygen therapy (HBOT) could reduce orthodontic relapse by altering the expressions of hypoxia-inducible factor (HIF)-1 messenger ribonucleic acid (mRNA), type I collagen (Col I), and matrix metalloproteinase-1 (MMP-1) in the gingival supracrestal fibers in rabbits.

**Materials and Methods** This study involved 44 male rabbits ( *Oryctolagus cuniculus* ) randomly divided into the normal group (K0), the orthodontic group without HBOT (K1), and the orthodontic group with HBOT (K2). Following orthodontic separation of the two upper central incisors, a retention phase and relapse assessment were performed. The HBOT was performed for a period of 2, 4, 6, 8, and 10 days after retention. HIF-1α transcription was assessed employing real-time polymerase chain reaction, whereas Col I and MMP-1 proteins were examined using immunohistochemistry. The orthodontic relapse was measured clinically using a digital caliper.

**Statistical Analysis** We used the one-way analysis of variance followed by Tukey's post hoc for multiple comparisons to measure differences between pairs of means; a *p-* value of 0.05 was considered statistically significant.

**Results** HBOT significantly increased the HIF-1α mRNA expression ( *p* = 0.0140), increased Col I ( *p* = 0.0043) and MMP-1 ( *p* = 0.0068) on the tensioned and pressured side of the gingival supracrestal fibers, respectively, and clinically decreased the relapse ( *p* = 3.75 × 10 ^−40^ ).

**Conclusion** HBOT minimizes orthodontic relapse by influencing HIF-1α expression, collagen synthesis (Col I), and degradation (MMP-1). This result suggests that HBOT has the potential to be used as an adjunctive method in the orthodontic retention phase.

## Introduction

Dental occlusion after orthodontic treatment basically has unstable potential that can move teeth back to their origin occlusion (relapse). Maintaining the position of corrected teeth is crucial and must be considered from the moment an orthodontic treatment plan is made. 1 Kaan and Madléna stated that retention after orthodontic intervention is as important as active therapy. 2

Even though the teeth have already been in their ideal position, the remodeling and reorganization of the supporting tissues of the teeth have not been finished. 3 4 Gingiva, unlike bone and periodontal membrane, is squeezed and retracted after orthodontic treatment. In compressed gingiva, the amount of elastic fibers grows considerably, mimicking compressed rubber, which once compressed returns to its original size.

In gingival homoeostasis, extracellular matrix component genes, which are involved in the synthesis and degradation of collagen, is constantly regulated. 5 Collagen synthesis occurs in gingiva under tension in the manner of tooth movement, as shown by elevated type I collagen (Col I) expression. Collagen degradation occurs in gingiva that is under pressure through the process of proteolysis by matrix metalloproteinase-1 (MMP-1). According to a molecular study, orthodontic mechanical stress increased the transcription rate of the Col I gene while decreasing the expression of the collagenase gene in gingival fibers. 6 Relapse may be associated with a prolonged disruption in the balance of gingival supracrestal collagen synthesis and degradation after orthodontic tooth movement. 5 6 7 8 To maintain appropriate tissue stability and avoid orthodontic relapse, an equilibrium between collagen catabolic and anabolic gingival fibers is necessary. 8

The study by Al Yami showed that the incidence of relapse could reach more than 50% of all patients treated orthodontically, 9 10 while Kaan and Madléna stated that relapse occurred in 70 to 90% of patients. 11 The results therefore illustrate how unstable orthodontic treatment is, as well as perplexity about the variables underlying this instability, and emphasizes the importance of informing patients about long-term treatment expectations. 12 Recently, many patients have become more cognizant of facial aesthetics. As a result, an increasing number of individuals seek orthodontic treatment. The benefits of therapy must be balanced against the risks, and any changes that occur after orthodontic treatment, including relapse, must be considered. 9

Various methods for reducing relapse have been developed, including relaxin injection, 13 low level laser therapy, 14 15 and circumferential supracrestal fibrotomy. 16 According to Meng et al, circumferential supracrestal fibrotomy is effective in reducing relapses but is invasive and has risks and contraindications. 17 In this study, we try to use hyperbaric oxygen therapy (HBOT), an uninvasive method that has the potential effects to promote the formation of new blood vessels and speed wound healing, to minimize orthodontic relapse.

HBOT is a procedure in which a patient inhales 100% oxygen while being subjected to an increased atmospheric pressure of up to 2 to 3 atmospheres (Atm) absolute for 1.5 to 2 hours, one to three times per day, depending on the indication. 18 19 20 21 The intracellular genesis of reactive oxygen and nitrogen species is a critical mechanism for HBOT. The role of reactive substances such as oxygen and nitrogen in this process is revealed through cell signaling transduction cascades. Nitrogen and reactive oxygen species combined cause cell damage, resulting in nitrative stress. 22 HBOT facilitates the delivery of oxygen to the tissues, and as a result, wound healing might be improved and patients' recovery times reduced. It promotes the formation of new blood vessels, resulting in increased tissue oxygenation, which can assist to stop infection and speed up wound healing. 20 23

The potential use of HBOT in orthodontic treatment seemed evident and supported by significant scientific evidences. However, the HBOT's effects got more investigated during active phase of orthodontic treatment. Salah and Eid demonstrated that HBOT might be useful in reducing tooth mobility during and after orthodontic treatment. 24 According to Prayogo et al, the interaction of HBOT and propolis gel affected the periodontal membrane width and the number of fibroblasts responsible for reducing orthodontic relapse risk. 25

Compared with other components of the periodontium, gingival tissue has not been yet investigated in studies highlighting the comprehensive interrelationship between orthodontics, periodontics, and HBOT. 26 HBOT offers some potential effects to be investigated in preventing orthodontic relapse but its role in reorganizing and repairing gingival fibers after orthodontic treatment is not fully understood.

The purpose of the present study was to discover how HBOT could reduce orthodontic relapse by altering the expressions of hypoxia-inducible factor (HIF)-1α messenger ribonucleic acid (mRNA), Col I, and MMP-1 in the gingival supracrestal fibers in rabbits.

## Materials and Methods

The experimental design and protocols were reviewed and approved by the Ethics Committee of the Health Research, Indonesian Naval Maritime Health Institute, Surabaya, Indonesia, under file number 01/EC/LKS/I/2021. This study was a true experiment conducted in rabbits. The sample size was calculated using the Federer formula, which is ( *t* – 1) ( *n* – 1) ≥ 15, where ( *t* ) is number of treatment groups and ( *n* ) is the number of samples per treatment group. According to these calculations, the minimal sample size is 33. The Higgins formula is then used to anticipate the sample dropped out, which *n* ' = *n* × 1/(1– *f* ), where ( *n* ) is the sample size before corrected, ( *f* ) is the estimated nonresponse (10%), and ( *n* ') is the sample size after correction. According to the calculation results, the sample size after the correction is 44 rabbits.

Objects were randomly divided into the blank normal group/K0 ( *n* = 4), the orthodontic group without HBOT/K1 ( *n* = 20), and the orthodontic group with HBOT/K2 ( *n* = 20). Using the lottery method, each member of the group was assigned a number and then was chosen at random. The randomly selected number represents the selected group member. Group K2 was further separated into five groups based on the HBOT given, as well as group K1 as control group to K2. The inclusion criteria were male rabbits, 6 to 7 weeks of age, body weight 150 to 160 g, healthy, showed no signs of decreased exploration behavior, and demonstrated tooth movement caused by orthodontic pressures. Exclusion criteria were suffering from a disease that prevented them from undergoing research experiments.

Each rabbits in groups K1 and K2 had their teeth moved distally by inserting an orthodontic elastic separator (Unistick colored ligature, American Orthodontics) between the upper left (U1L) and right (U1R) central incisors. The force generated by the separator was 0.29 g/cm ^2^ . After 14 days of separation, the separator was removed and a piece of stainless steel wire 0.018” was attached to the teeth surface with the assistance of a flowable light-cured composite (D-Tech Compo Flo Light Cure) to keep the teeth in place (retention).

After 7 days of retention, the retainer was removed and the distance between the most distal points of upper left to right central incisors at ⅓ incisal level was measured using a digital caliper (Digimatic Caliper Mitutoyo, Model No. CO-6” ASX, Code No. 500–196–30, Serial No. A19114651). The method was performed by two independent individuals who had passed the intra- and interexaminer reliability assessments in a preliminary research which each examiner measured the same objects two times at 8 a.m. and 4 p.m. The examiner was considered reliable when he provided ≥ 0.700 consistent results for the same measurements.

**Fig. 1:**
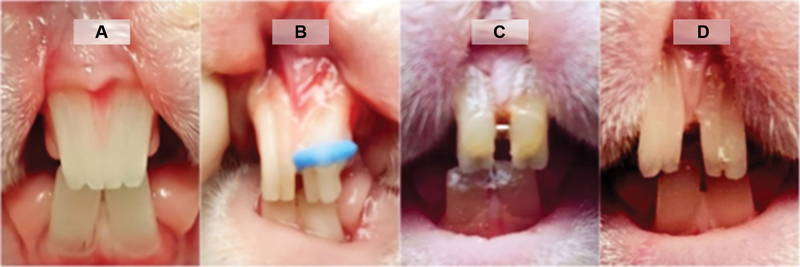
Tooth distance. ( **A** ) Before separation. ( **B** ) During separation. ( **C** ) Retention period. ( **D** ) Relapse observation.

Objects in group K2 exclusively got HBOT in animal hyperbaric chamber using protocol by Hart Kindwall (Kindwall's table) 27 for the monoplace chamber which includes the delivery of 100% oxygen at 2.4 Atm for 90 minutes without air breaks. HBOT was conducted at 7 a.m. for 10 consecutive days. The distance of U1L to U1R was measured on days 2, 4, 6, 8, and 10 after HBOT, just before termination to know the amount of tooth relapse. The measurement was also performed for group K1 on the same days as group K2. Fig. 1 demonstrates the sequences of rabbits' teeth movements in this study.

The rabbits were given intracardiac injections of acepromazine concentrate (15 mg/kg body weight) and ketamine (20 mg/kg body weight). All the procedures were performed by a veterinarian in a veterinary hospital (Faculty of Veterinary Medicine, Airlangga University, Surabaya, Indonesia) according to ethical guidelines for research in animal science. 28 After confirming death, gingival tissue samples were taken and then put into a container filled with formalin for immunohistochemical preparations and into a microtube containing RNAlater for real-time polymerase chain reaction (RT-PCR) examination.

### RT-PCR for HIF-1α Expression

The RT-PCR procedures were performed in the Integrated Research Laboratory, Faculty of Dentistry, Universitas Padjadjaran and Biomolecular Laboratory, Faculty of Medicine, Universitas Padjadjaran, Bandung, Indonesia. The procedures comprised RNA extraction (Zymo Research Quick-RNA MiniprepPlus Kit) and RT-PCR preparation (SensiFAST SYBR No-ROX). In this study, the primers constructed were (Rabbit) HIF-1α 5′-CAGCAGCCAGATGATCGTACA-3′ (forward) and 5′-TCCATTGATTGCCCCAGCAG-3′ (reverse) with 149 base pair (bp). Housekeeping primers were (Rabbit) GAPDH 5′-GAATCCACTGGCGTCTTCAC-3′ (forward) and5′-CGTTGCTGACAATCTTGAGAGA-3′ (reverse) with 160 bp.

To assess mRNA HIF-1 expression data, the relative quantification (2 ^−ΔΔCT^ method) 29 was utilized, comparing the target transcript PCR signal inside a therapy group with that of a different sample, such as a control sample. In a time-course study, relative quantification indicates the difference in the transcription of the target gene compared with a certain reference group, which could be a control treatment or a sample at time zero.

### Immunohistochemistry for Col I and MMP-1

The immunohistochemistry examination was performed in the Biochemistry Laboratory of Experimental Animal Unit, Faculty of Veterinary Medicine, Airlangga University, Surabaya, Indonesia. The procedures were in accordance with the instructions included with the immunohistochemical test kit, and stained with primary Col I polyclonal antibodies Cat. No. GTX26308 (GeneTex International Corporation) and mouse anti-human MMP-1-FITC; Clone SB12e Cat. No. 12011 (SouthernBiotech, Birmingham, Alabama, United States). A modified semiquantitative immunoreactive score of Remmele was used to analyze the level of Col I and MMP-1, which was derived by multiplying the score of immunoreactive transfected cells or regions by the color intensity score of immunoreactive cells or areas. At 400× magnification, data from each sample was collected and averaged from five different view fields. All of these tests were performed employing a conventional Nikon Eclipse light microscope system that includes a 300-megapixel DS Fi2 digital camera and Nikkon Image System software for image processing. For all statistical tests, SPSS software version 20.0 was utilized.

### Orthodontic Relapse

**Fig. 2:**
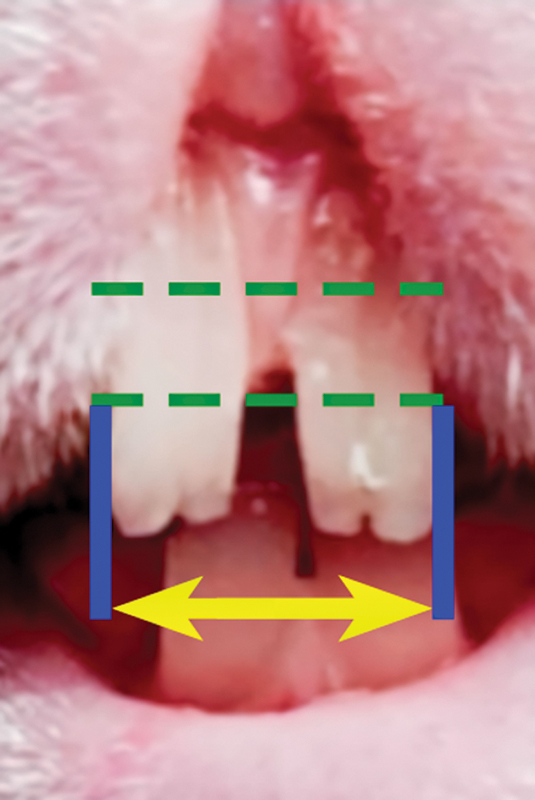
Tooth distance measurement. Distance between the most distal points of upper left to right central incisors (U1L – U1R) at ⅓ incisal level.

Tooth distance was measured as the distance between the most distal points of each upper central incisor at ⅓ incisal level at a certain time period, that is, before tooth movement, after tooth movement, at the end of the retention, and after retention/at the end of the observation. Measurements were performed by two independent calibrated examiners at 7 a.m. at every determined days. Relapse was defined as the difference between the distance at the end of the retention minus the distance at the end of the observation ( Figs. 1 and 2 ).

## Results

**Fig. 3:**
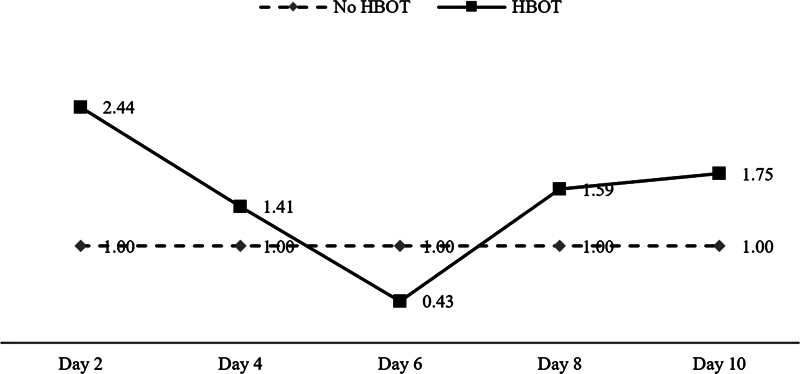
Expression of hypoxia-inducible factor (HIF)-1 messenger ribonucleic acid (mRNA) between groups K2 (hyperbaric oxygen therapy [HBOT]) and K1 (without HBOT).

Table 1 displays the descriptive statistics of the RT-PCR evaluation for HIF-1 mRNA expression in groups K2 and K1. Statistical analysis of the similarity test showed a value of *p* = 0.0140, demonstrating a significant difference in HIF-1 mRNA expression rates comparing groups K2 and K1 ( Table 2 ). The difference in HIF-1 mRNA levels between groups K2 and K1 is shown in Fig. 3 .

**Table 1:** Descriptive of HIF-1α mRNA expression in K1 and K2 groups

	Group	ΔCT (Ct target gene – Ct GAPDH)	ΔΔCT (ΔCT treatment – ΔCT control)	Gene expression (2 ^ ^(−ΔΔCT)^ )
HBOT	K2.1	0.83	–1.29	2.44
	K2.2	3.96	–0.50	1.41
	K2.3	6.58	1.23	0.43
	K2.4	2.33	−0.67	1.59
	K2.5	2.20	−0.81	1.75
No HBOT	K1.1	2.12		
	K1.2	4.45		
	K1.3	5.35		
	K1.4	3.01		
	K1.5	3.01		

Abbreviations: GAPDH, glyceraldehyde 3-phosphate dehydrogenase; HBOT, hyperbaric oxygen therapy; HIF, hypoxia-inducible factor; mRNA, messenger ribonucleic acid.

**Table 2:** Analysis of similarities of HIF-1α mRNA expression between K2 and K1 groups

	K2	K1
Day 2	2.441	1
Day 4	1.409	1
Day 6	0.428	1
Day 8	1.594	1
Day 10	1.753	1
*n*	20	20
Mean	1.53	1
SD	0.73	0
*t* -Test	2.28	
*p* -Value	0.0140 a	
	Sign	

Abbreviations: HIF, hypoxia-inducible factor; mRNA, messenger ribonucleic acid; SD, standard deviation.

a *α* = 0.05.

**Fig. 4:**
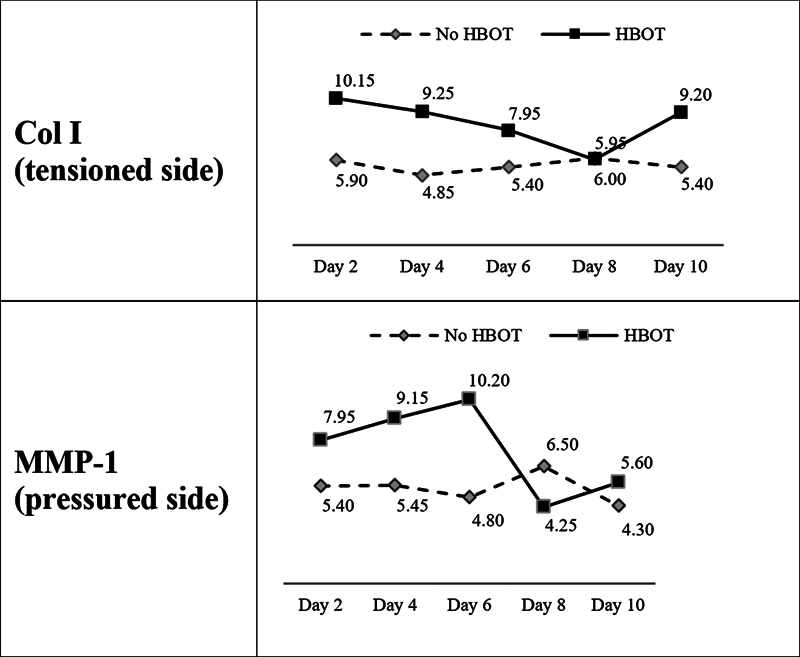
Expressions of type I collagen (Col I) protein (tensioned side) and matrix metalloproteinase-1 (MMP-1) (pressured side).

The descriptive results of the immunohistochemical examination of Col I expression in the tensioned side and MMP-1 in the pressured side are presented in Table 3 . The analysis of variance (ANOVA) test showed significant differences between the means of 11 groups in expressing Col I and MMP-1 with *p* = 0.0043 and *p* = 0.0068, respectively, at the level of significance *α* = 0.05 ( Table 4 ). Furthermore, to uncover specific differences between the groups, post hoc tests were performed with independent *t* -test. Post hoc test ( Table 5 ) showed that Col I expression in the tensioned side increased significantly in group K2–1 compared with K1–1 ( *p* = 0.0054), group K2–2 compared with K1–2 ( *p* = 0.0041), and K2–5 compared with K1–5 ( *p* = 0.0119). MMP-1 expression in the pressurized side increased significantly in groups K2–2 over K1–2 ( *p* = 0.0075) and K2–3 versus K1–3 ( *p* = 0.0027) ( Table 6 ). The changes in Col I and MMP-1 expressions are shown in Fig. 4 .

**Table 3:** Descriptive scores for Col I (tensioned side) and MMP-1 (pressured side)

	Group	Col I (tensioned side)			MMP-1 (pressured side)		
		Mean	*n*	SD	Mean	*n*	SD
No HBOT	** K1–1**	**5.90**	**4**	**1.291**	**5.40**	**4**	**2.688**
	K1–2	4.85	4	0.719	5.45	4	1.865
	K1–3	5.40	4	3.702	4.80	4	3.521
	K1–4	6.00	4	0.730	6.50	4	3.675
	K1–5	5.40	4	1.541	4.30	4	1.013
HBOT	K2–1	10.15	4	1.700	7.95	4	1.370
	K2–2	9.25	4	1.012	9.15	4	2.199
	K2–3	7.95	4	1.025	10.20	4	1.470
	K2–4	5.95	4	4.139	4.25	4	3.087
	K2–5	9.20	4	1.405	5.60	4	1.143

Abbreviations: Col I, type I collagen; HBOT, hyperbaric oxygen therapy; MMP-1, matrix metalloproteinase-1; SD, standard deviation.

**Table 4:** ANOVA table for Col I (tensioned side) and MMP-1 (pressured side)

	Col I (tensioned side)					MMP-1 (pressured side)				
Source	SS	df	MS	*F*	*p* -Value	SS	df	MS	*F*	*p* -Value
Treatment	135.562	10	13.5562	3.33	0.0043 a	172.709	10	17.2709	3.11	0.0068 a
Error	134.300	33	4.0697			183.440	33	5.5588		
Total	269.862	43				356.149	43			

Abbreviations: ANOVA, analysis of variance; Col I, type I collagen; df, degrees of freedom; MMP-1, matrix metalloproteinase-1; MS, mean square; SS, sum of squares.

a *α* = 0.05.

**Table 5:** Post hoc analysis for Col I (tensioned side)

	K1–2	K1–3	K1–5	K1–1	K2–4	K1–4	K0	K2–3	K2–5	K2–2	K2–1
K1–2											
K1–3	0.702										
K1–5	0.702	1.000									
K1–1	0.467	0.728	0.728								
K2–4	0.446	0.702	0.702	0.972							
K1–4	0.426	0.677	0.677	0.945	0.972						
K0	0.089	0.181	0.181	0.317	0.334	0.351					
K2–3	0.037*	0.083	0.083	0.160	0.170	0.181	0.677				
K2–5	0.004**	0.012*	0.012*	0.027*	0.029*	0.032*	0.204	0.387			
K2–2	0.004**	0.011*	0.011*	0.025*	0.027*	0.029*	0.192	0.369	0.972		
K2–1	0.001**	0.002**	0.002**	0.005**	0.006**	0.006**	0.058	0.133	0.510	0.532	

Abbreviation: Col I, type I collagen.

**Table 6:** Post hoc analysis for MMP-1 (pressured side)

	K0	K2–4	K1–5	K1–3	K1–1	K1–2	K2–5	K1–4	K2–1	K2–2	K2–3
K0											
K2–4	0.929										
K1–5	0.905	0.976									
K1–3	0.677	0.744	0.766								
K1–1	0.441	0.495	0.514	0.721							
K1–2	0.424	0.477	0.495	0.699	0.976						
K2–5	0.375	0.424	0.441	0.634	0.905	0.929					
K1–4	0.159	0.186	0.196	0.315	0.514	0.533	0.593				
K2–1	0.027*	0.033*	0.036*	0.068	0.136	0.143	0.168	0.391			
K2–2	0.005**	0.006**	0.006**	0.014*	0.031*	0.033*	0.041*	0.121	0.477		
K2–3	0.001**	0.001**	0.001**	0.003**	0.007**	0.007**	0.009**	0.033*	0.186	0.533	

Abbreviation: MMP-1, matrix metalloproteinase-1.

Note: *α* = 0.05.

**Fig. 5:**
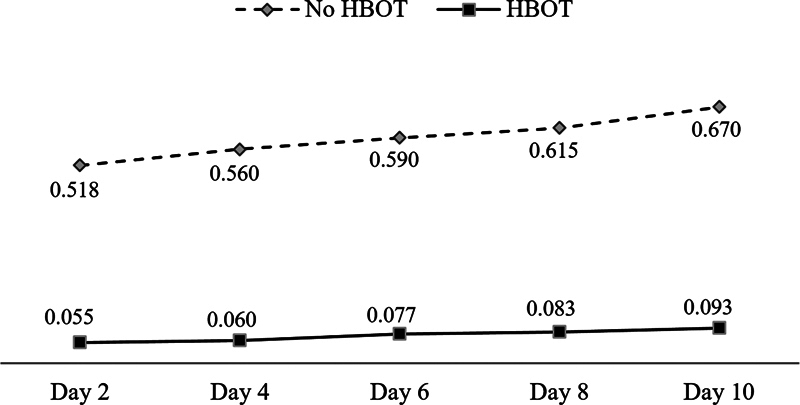
Tooth relapse in K1 groups (no hyperbaric oxygen therapy [HBOT]) and K2 groups (HBOT) in millimeters.

Table 7 shows a description of the amount of relapse between subjects in groups K1 and K2. The ANOVA test revealed that the amount of relapse differed significantly between the 10 groups, with *p* = 3.75 × 10 ^−40^ ( *p* > 0.05) ( Table 8 ). The post hoc test revealed that relapse was considerably lower in all K2 groups than in K1 ( Table 9 ). Fig. 5 depicts the amounts of tooth relapse in the two groups.

**Table 7:** Descriptive of the amount of relapse

	Group	Mean	*n*	SD
No HBOT	K1–1	0.518	4	0.0171
	K1–2	0.560	4	0.0082
	K1–3	0.590	4	0.0082
	K1–4	0.615	4	0.0129
	K1–5	0.670	4	0.0183
HBOT	K2–1	0.055	4	0.0058
	K2–2	0.060	4	0.0082
	K2–3	0.077	4	0.0096
	K2–4	0.083	4	0.0096
	K2–5	0.093	4	0.0096

Abbreviations: HBOT, hyperbaric oxygen therapy; ; SD, standard deviation.

**Table 8:** ANOVA test for the amount of relapse

Source	SS	df	MS	*F*	*p* -Value
Treatment	2.7295	9	0.30328	2332.94	3.75E-40 a
Error	0.0039	30	0.00013		
Total	2.7334	39			

Abbreviations: ANOVA, analysis of variance; df, degrees of freedom; MS, mean square; SS, sum of squares.

a *α* = 0.05.

**Table 9:** Post hoc analysis for the amount of relapse

	K2–1	K2–2	K2–3	K2–4	K2–5	K1–1	K1–2	K1–3	K1–4	K1–5
K2–1										
K2–2	0.5									
K2–3	0.0**	0.0*								
K2–4	0.0**	0.0**	0.5							
K2–5	0.0**	0.0**	0.1	0.2						
K1–1	3.2 × 10 ^−32^ **	4.4 × 10 ^−32^ **	1.4 × 10 ^−31^ **	1.9 × 10 ^−31^ **	3.9 × 10 ^−31^ **					
K1–2	2.3 × 10 ^−33^ **	3.1 × 10 ^−33^ **	8.9 × 10 ^−33^ **	1.2 × 10 ^−32^ **	2.3 × 10 ^−32^ **	1.1 × 10 ^−5^ **				
K1–3	4.1 × 10 ^−34^ **	5.5 × 10 ^−34^ **	1.5 × 10 ^−33^ **	2.0 × 10 ^−33^ **	3.6 × 10 ^−33^ **	5.1 × 10 ^−10^ **	.0**			
K1–4	1.0 × 10 ^−34^ **	1.4 × 10 ^−34^ **	3.6 × 10 ^−34^ **	4.8 × 10 ^−34^ **	8.4 × 10 ^−34^ **	4.6 × 10 ^−13^ **	1.4 × 10 ^−7^ **	0.0**		
K1–5	6.5 × 10 ^−36^ **	8.3 × 10 ^−36^ **	2.0 × 10 ^−35^ **	2.5 × 10 ^−35^ **	4.2 × 10 ^−35^ **	3.2 × 10 ^−18^ **	2.1 × 10 ^−18^ **	5.5 × 10 ^−11^ **	1.4 × 10 ^−7^ **	

Note: *α* = 0.05.

## Discussion

The purpose of the present study was to discover how HBOT could reduce orthodontic relapse by altering the expressions of HIF-1 mRNA, Col I, and MMP-1 in the gingival supracrestal fibers in rabbits. This study used a specific HBOT protocol in the monoplace chamber because it was not possible for the experimental animals to inhale pure O _2_ using a mask and to do air breaks for a certain period. Monoplace chambers are designed without the ability to provide air breaks. Treatment tables within these limits have been tested and found to be effective in most cases with minimal risk of oxygen toxicity. 27

This present study found that HBOT significantly increases HIF-1 mRNA expression. This result is quite different with some other studies. We concluded that the different results were caused by different objects, the HBOT protocol (the amount of hyperbaric and oxygen concentration, duration, and the number of sessions) and target tissues expressing HIF-1α.

Numerous studies on the effect of HBOT on HIF-1 expression have been published, but the results were varied. Studies by Zhang et al, 30 Sunkari et al, 31 Růžička et al, 32 and Lindenmann et al 33 concluded that HBOT could increase HIF-1α expression. Meanwhile, Sun et al, 34 Zhang et al, 35 Chen et al, 36 and Sari et al 37 mentioned that HBOT suppressed the expression of HIF-1α.

The contradiction can be seen not only in the HIF-1α but also in several other biomarkers. This reflects that HBOT tends to stabilize and restore physiological equilibrium rather than enhance or inhibit a biological mechanism. Nonetheless, applying HBOT in clinical settings has demonstrated beneficial effects on tissue regeneration via mechanisms such as stem cell homoeostasis, oxidative stress response, inflammation, tissue remodeling, angiogenesis, cell adhesion/contact, regeneration, proliferation, differentiation, and apoptosis.

The “hyperoxic-hypoxia paradox” theory put by Hadanny and Efrati 38 may justify the contradiction. Hypoxia is a natural trigger for changes in mitochondrial metabolism through increased levels of HIF, vascular endothelial growth factor, sirtuins, and proliferation and migration of progenitor cells. Intermittent hyperoxia can be generated via HBOT. Fluctuations in oxygen levels can initiate cellular cascades that provide a basis for intermittent hyperoxia procedures to promote tissue regeneration while avoiding the negative consequences of hypoxia. Variations in oxygen supply have a bigger impact on HIF expression than persistent hypoxia or hyperoxia. Within this theory, cells interpret a shift from normoxia to hypoxia or a shift back to normoxia after hyperoxia exposure as oxygen deprivation and promote the production of HIF-1-regulated genes. Unfortunately, the precise time frame for repeated administration and inspired oxygen levels are not entirely established.

In this study, we examined the HBOT effects to reduce orthodontic relapse in three consecutive phases, that is, molecular phase indicated by expression of HIF1-α mRNA (RT-PCR), protein phase by the expression of Col I and MMP-1 (immunohistochemistry), and clinical phase by the amount of orthodontic relapse. Stegen et al 39 mentioned that prolonged HIF-1α interfered with cellular bioenergetics and biosynthesis. Decreased glucose oxidation results in an energy deficit, which limits proliferation, activates the unfolded protein response, and reduces collagen synthesis. However, enhanced glutamine flux increases α-ketoglutarate levels, which in turn increases collagen proline and lysine hydroxylation. This metabolically regulated collagen modification renders the matrix more resistant to protease-mediated degradation. Thus, inappropriate HIF-1α signaling results in dysplasia caused by collagen overmodification, an effect that may also contribute to other extracellular matrix-related diseases such as cancer and fibrosis.

The gingival tissue is constantly exposed to physical forces. Mechanical stimulation regulates connective tissue equilibrium, and persistent mechanical stimulation processes can alter extracellular matrix structure and cell activity. Human gingival fibroblasts (HGFs) are in charge of extracellular matrix formation and breakdown, as well as proteolytic enzyme secretion. 40 41 Mechanical stress applied to HGF grown on three-dimensional poly(lactic-co-glycolic acid) (3D PLGA) scaffolds increased Col I gene expression while decreasing MMP-1 synthesis via the transforming growth factor signaling pathway. 42

By evoking a biological reaction, orthodontic force, as an external mechanical stimulation, not just moves the teeth but also restores balance in the periodontal tissues. Changes in gingival fibroblast proliferation by mechanical forces reflect the establishment of a new balance. In normal tissue, collagen synthesis and degradation are required for the constant remodeling of connective tissue. Degradation is essential for tissue formation and repair, as well as abnormal processes such as cancer and metastasis.

The most essential method of connective tissue remodeling is intracellular degradation of collagen, which includes fibril identification by binding to fibroblast receptor molecules, partial breakdown into smaller fibrils, the formation of phagolysosomes, and fibril elimination by lysosomal enzymes. Fibroblasts, inflammatory cells, and tumor cells secrete MMPs, which cause extracellular disintegration. 43 44 45 Redlich et al found that two different processes occur in the gingiva after orthodontic force transduction. 8 The damaged collagen fibers activate collagen genes on the tensioned side while collagenase is produced on the pressured side. The authors believe that this phenomenon may explain the decrease in relapse rates after HBOT. Furthermore, the behavior of changes in Col I as well as MMP-1 protein expression in this study appears to be in accordance with the results of Nan et al, 46 who discovered that the proliferation of HGF cells cultured in 3D PLGA scaffolds increased significantly and peaked after 24 and 48 hours of mechanical stimulation. Cell proliferation was halted after 72 hours, and the number of cells steadily decreased.

Several limitations of the current study that were beyond the researchers' control and were unable to be addressed in this research might be identified. Studies that specifically evaluate the effect of HBOT solely on the gingival supracrestal fibers can be conducted on fibroblast cultures. The addition of a control group that only receives HBOT without orthodontics may allow researchers to better explain the mechanism of HBOT in minimizing orthodontic relapse. This current study could lead to future investigation exploring different possible pathways of HBOT's effect in the regeneration of gingival supracrestal fibers, to reduce orthodontic relapse. Furthermore, the findings of this study must be evaluated in larger experimental animals, such as nonhuman primates, before applying in human clinical trials.

## Conclusion

Within the limitations of the present study, we conclude that:

1. HBOT increases HIF-1α mRNA expression in the gingival supracrestal fibers after orthodontic tooth movement.
2. HBOT affects collagen synthesis by increasing Col I protein expression on the tensioned side of the gingival supracrestal fibers after orthodontic tooth movement.
3. HBOT influences collagen degradation by increasing MMP-1 protein expression on the pressured side of the gingival supracrestal fibers after orthodontic tooth movement.
4. As an adjunctive therapy, HBOT minimizes relapse after orthodontic tooth movement.
